# Bacterial and Fungal Community Composition and Functional Activity Associated with Lake Wetland Water Level Gradients

**DOI:** 10.1038/s41598-018-19153-z

**Published:** 2018-01-15

**Authors:** Yantian Ma, Jinqian Li, Juan Wu, Zhaoyu Kong, Larry M. Feinstein, Xia Ding, Gang Ge, Lan Wu

**Affiliations:** 1 Key Laboratory of Poyang Lake Environment and Resource, Ministry of Education, and School of Life Sciences, Nanchang University, Nanchang, 330022 China; 2 Key Laboratory of Aquatic Animal Resources and Utilization of Jiangxi, Nanchang University, Nanchang, 330022 China; 3 University of Maine at Presque Isle, Presque Isle, Maine, USA

## Abstract

The water regime is often the primary force driving the evolution of freshwater lakes, but how soil microbes responded to this process is far from understood. This study sampled wetland soils from a shallow lake that experienced water regime changes, Poyang Lake of China, to explore the features of bacterial and fungal community in response to water level changes. The soil physicochemical properties, T-RFLP based community structures and soil activities (including basal respiration, microbial biomass and enzymes) were all determined. Soil microbial eco-function was captured by testing the carbon metabolism with Biolog-Ecoplate. The results showed remarkable influence of the water level gradients on the soil physicochemical properties, microbial community structures and soil activities. However, the carbon utilization profile exhibited weak connections with the environmental variables and microbial community structures (*p* > 0.05). The microbial activities were significantly correlated with both bacterial and fungal community structures. Our results also emphasized the ascendant role of the deterministic process in the assemblages of microbial community structures and functions in wetland. In conclusion, this study revealed the discrepancy between community structures and eco-functions in response to water level gradients, and a relatively stable eco-function helped to maintain the ecosystem function of wetland from a long-term perspective.

## Introduction

Freshwater lakes provide important ecological and economic value, including local climate regulation, a habitat that supports biodiversity, and resources for economic development. Lake wetlands are an important functional habitat that often contain the highest productivity and microbial activity in the lake region. They are also regions susceptible to environmental disturbances, including changes in hydrology, water quality, and vegetation^1^. However, the important ecological contributions to biogeochemical cycling by lake wetlands have been underestimated in a global view due to their small proportional area^2–4^. Because of these important contributions, it is crucial to understand how environmental disturbances impact wetland function.

In recent years, global climate change and anthropogenic activities, including the construction of hydropower stations and dams, aquaculture, and agricultural reclamation, have profoundly impacted the water regime of the lake ecosystem. Water regime changes may strongly affect the wetland microbial composition as well as functional activity in terms of biogeochemical cycling and energy flux^5^. Altered hydrological and physicochemical characteristics of the waters and soils have been documented to affect the plant, animal, and microbial communities^6–9^. Many studies have described shifts in the soil microbial community composition or community functional activity, but most did not investigate changes in the community composition and function together^10–13^; thus, it remains unclear to what extent community structure determines a functional profile in the different environments^14,15^. Moreover, shifts in the community composition do not always cause changes in the community functional processes^16^. When facing environmental or anthropogenic disturbances, the microbial community structure and function may respond separately or concurrently^17,18^. Studies that quantify changes in the community structure and function in response to disturbance have been few up to now, and much work is needed to understand these changes.

In addition to the uncertain link between the microbial community structure and function, there is considerable debate about what drives microbial community assembly. The most frequently proposed mechanisms concerning community assembly and the spatial distribution of soil microbial communities are niche-related (deterministic) and neutral (stochastic) processes^19–23^. These processes may occur at different scales^24^, and many surveys showed the combined effects of both theories^25^. pH^26^, soil moisture^27^, phosphate and the C:N ratio^28^ were all important drivers in the deterministic process, while the dispersal limitation of the stochastic processes were noted in *Actinobacteria*^29^, AM fungi^21^, and aquatic bacterial communities^30^. While studies have quantified the relative contributions of niche and neutral processes to community assembly, very few have compared these processes in variable habitats, such as lake wetlands that experience periodic fluctuations in hydrology. In the highly complex and dynamic wetland lake ecosystem, there is a lack of understanding regarding the link between community structure and function and the influence of the niche or neutral community assembly processes. Increasing our understanding of these factors may help with wetland management and preservation.

The objective of this study was to investigate how water level gradients (or hydroperiods) affect the wetland microbial community composition and community ecological functions. We also wanted to quantify the relative influence of the deterministic and stochastic processes in structuring the spatial distribution patterns of the wetland microbial communities. We hypothesized that the microbial functions would be more stable than the composition in coping with the water level gradients and that the deterministic process would play a greater role than the stochastic process in structuring the microbial community assembly. We sampled wetland soils with different water level gradients from the lakeside of Poyang Lake, the largest freshwater lake of China^31^. Soil physicochemical properties, microbe-related soil activities (enzymes, biomass and respiration), bacterial and fungal community composition and Eco-plate carbon utilization profiles were all measured and analysed. The variance partitioning method was also used to determine the impact of the deterministic and stochastic processes in this study.

## Results

### The impact of water gradients on soil properties and vegetation communities

Water level gradients were associated with differences in several shoreline wetland soil properties, including SM (soil moisture), TOC (total organic carbon) and TP (total phosphorus). The SM decreased from the H1 plot to the H6 plot, along with an increase in the water level gradients. Meanwhile, the soil pH values showed significant differences between the near water plots (H1-H3) and remote water plots (H4-H6) (*p* < 0.05). However, most soil nutrient-related parameters failed to exhibit a clear, constant gradient, including AFDM, TN and NO_3_-N (Table 1). The TOC content divided all the tested plots into two groups; H1-H4 had less TOC than the H5-H6 group. The H1 plot persisted in having a very high NH_4_-N content (23.54 ± 1.00 mg/kg), which was approximately 4–8 times higher than that in the other plots (3.01–6.24 mg/kg).

**Table 1:** Environmental variables and vegetation characteristics from the different plots of Lake Poyang.

Plots	Water level heights (WH, m)	Hydroperiod (days)	SM/%	pH	AFDM/%	TOC (g/kg)	TN (g/kg)	TP (g/kg)	NH_4_-N (mg/kg)	NO_3_-N (mg/kg)	Vegetation		
											Vegetation species	Vegetation abundance	Predominant species
H6	4.80	30–35	26.25 ± 0.35 e	5.02 ± 0.05 d	7.21 ± 0.32 b	15.54 ± 0.36 b	1.63 ± 0.08 ab	0.24 ± 0.01 bc	3.95 ± 0.71 b	1.13 ± 0.48 a	5.00 ± 0.00 a	13.00 ± 0.58 a	*Cynodon dactylon* (Linn.) Pers; *Carex cinerascens* Kukenth; *Tulipa edulis* (Miq.) Baker; *Artemisia selengensis*; *Verbena officinalis* Linn.; *Potentilla limprichtii* J Krause
H5	3.17	75–90	28.45 ± 0.21 d	5.03 ± 0.02 d	8.18 ± 0.33 a	19.02 ± 0.76 a	1.87 ± 0.47 a	0.19 ± 0.01 c	5.57 ± 0.84 b	0.73 ± 0.35 a	5.00 ± 0.00 a	10.33 ± 0.33 a	*Triarrhena lutarioriparia* L. Liou; *C. cinerascens*; *Ranunculus polii* Franch. ex Hemsl. et Hemsley; *T. edulis*; *P. limprichtii*
H4	1.76	110–125	28.87 ± 0.16 d	5.24 ± 0.04 d	6.90 ± 0.13 b	9.76 ± 0.29 cd	1.32 ± 0.13 ab	0.29 ± 0.02 b	3.10 ± 0.57 b	0.66 ± 0.12 a	3.33 ± 0.33 b	6.67 ± 0.33 b	*C. cinerascens*; *R. polii*; *T. edulis*; *P. limprichtii*
H3	0.51	160–180	31.64 ± 0.64 c	6.48 ± 0.05 a	6.63 ± 0.01 b	9.08 ± 0.38 d	0.62 ± 0.14 b	0.37 ± 0.02 a	3.01 ± 0.13 b	0.91 ± 0.08 a	3.67 ± 0.33 b	6.67 ± 1.76 b	*C. cinerascens*; *Polygonum hydropiper* Linn.; *Rumex acetosa* Linn.; *Alopecurus aequalis* Sobol.; *R. polii*
H2	0.25	180–200	37.10 ± 0.37 b	6.07 ± 0.10 b	6.29 ± 0.16 b	10.76 ± 0.54 cd	1.09 ± 0.27 ab	0.29 ± 0.03 b	6.24 ± 1.01 b	0.84 ± 0.15 a	0 c	0 c	
H1	0	365	40.40 ± 0.29 a	5.81 ± 0.07 c	6.79 ± 0.23 b	11.68 ± 0.41 c	1.48 ± 0.16 ab	0.27 ± 0.01 b	23.54 ± 1.00 a	0.98 ± 0.18 a	0 c	0 c	

Water level heights (WH), the height above the water level; SM, soil moisture; AFDM, ash free dry mass; TOC, total organic carbon; Vegetation species, the mean number of plant species per plot; Vegetation abundance, the total number of plants per plot; All data are represented by the mean ± s.d., significant differences are marked with different letters in each column (*p* < 0. 05, n = 3).

The flooding hydroperiod differed among the plots, and the lowest H1 plots were submerged all year, whereas the highest H6 plots were only submerged for approximately one month (Table 1). The vegetation distribution was quite distinct. All plots could be divided into three groups based on the species number and abundance, the naked group (H1-H2), sparse group (H3-H4) and dense group (H5-H6). Greater plant growth (H5 and H6) was associated with drier conditions, which were observed in the plots furthest from the lake surface, while the long-term flooding conditions of the H1 and H2 plots repressed plant growth.

### Microbial community profiles from T-RFLP

**Figure 1:**
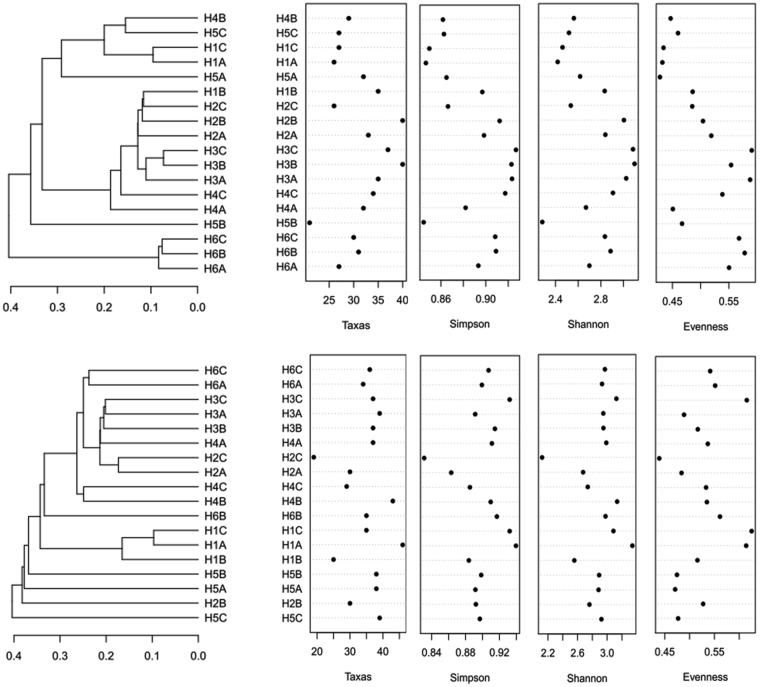
The H-clust analysis of the bacterial (upper) and fungal (lower) T-RFLP profiles and the corresponding diversity index from each plot. The A, B and C after the plot name indicate the triplicates.

A total of 388 bacterial TRFs and 420 fungal TRFs were detected by T-RFLP, and approximately 80% of the total TRFs remained for the bacterial and fungal community after quality control, including 147 bacterial TRFs and 206 fungal TRFs, respectively. The cluster analysis of the bacterial community revealed three different groups of study plots: the H6 plot was primarily separated from the others, and then, the H1 and H5 plots were also separated from the H2, H3 and H4 plots (Fig. 1). However, the clustering result of the fungal community was inconsistent with that of the bacterial community, and the H5 and H1 plots were distinguished from the H2, H3, H4 and H6 plots. Four of the community diversity indexes were evaluated, but no unified tendency was extracted (Fig. 1 and Table S1).

### Microbial-related activities and carbon utilization of soil microbial communities

The soil enzyme activity of BX, BG, NAG and PHOS varied among the plots, and the distribution profiles of each enzyme also differed. Generally, H5 and H6 plots had higher activities than the other plots for all the enzymes except for BG. The activities of BX, NAG and PHOS were highest in the H5 plot (*p* < 0.05), and their highest values were 40.23 ± 10.53, 54.97 ± 5.71 and 875.78 ± 90.52 μmol h^−1^ g^−1^, respectively. The H1, H2 and H5 plots all had higher BG activity than the other plots, including H6 (Table 2). The H5 and H6 plots had significantly higher microbial biomass carbon (MBC) and nitrogen (MBN) than the other plots (*p* < 0.05). The MBC contents were similar among H1-H4, but the MBN contents exhibited a profile of H1 > H4 > H3 > H2. The comparison of the soil basal respiration (BR) only distinguished the H2 plot with the lowest value. The distribution profile of qCO_2_ (h^−1^) was similar to that of MBC; the H5 and H6 plot values were higher than those of the H1-H4 plots (Table 2). The microbial carbon (MBC/TOC) and nitrogen (MBN/TN) were both higher in the H5 and H6 plots, and H1-H4 were similar except for H2, which had the lowest microbial nitrogen content at 0.14 ± 0.09% (Table 2).

**Table 2:** The soil enzymes, microbial biomass and respiration features among the plots in this study.

Plots	BX (ηmol/h/g)	BG (ηmol/h/g)	NAG (ηmol/h/g)	PHOS (ηmol/h/g)	MBC (mg/kg)	MBN (mg/kg)	BR (mg/kg/h)	qCO2 (h^−1^)	Microbial C (%)	Microbial N (%)
H6	17.33 ± 3.13 a	25.67 ± 3.17 a	19.09 ± 1.32 a	453.81 ± 8.22 b	211.47 ± 12.05a	22.15 ± 0.90a	9.95 ± 0.78ab	0.05 ± 0.01b	1.35 ± 0.09a	1.36 ± 0.05a
H5	40.23 ± 10.53 b	70.44 ± 11.09 b	54.97 ± 5.71 b	875.78 ± 90.52 c	235.17 ± 19.90a	22.40 ± 1.82a	12.28 ± 0.13a	0.05 ± 0.004b	1.25 ± 0.27a	1.37 ± 0.67a
H4	3.90 ± 0.93 a	25.00 ± 6.38 a	13.06 ± 2.52 a	170.07 ± 17.19 a	71.13 ± 4.41b	5.63 ± 0.37e	10.32 ± 0.52ab	0.15 ± 0.01a	0.73 ± 0.06b	0.43 ± 0.04b
H3	3.26 ± 0.96 a	26.52 ± 5.40 a	15.77 ± 3.22 a	86.50 ± 22.42 a	50.12 ± 4.16b	5.30 ± 0.12c	10.47 ± 0.93ab	0.21 ± 0.02a	0.56 ± 0.07b	0.95 ± 0.42ab
H2	2.57 ± 0.44 a	47.07 ± 5.65 ab	14.50 ± 1.09 a	93.97 ± 20.57 a	46.90 ± 10.29b	1.33 ± 0.12d	8.58 ± 0.44b	0.20 ± 0.04a	0.44 ± 0.09b	0.14 ± 0.09b
H1	3.08 ± 0.69 a	54.92 ± 13.79 ab	14.30 ± 3.68 a	66.22 ± 13.48 a	83.33 ± 6.63b	9.94 ± 1.08b	11.22 ± 1.09ab	0.13 ± 0.01a	0.72 ± 0.08b	0.68 ± 0.13ab

Microbial C/N is represented by MBC/TOC and MBN/TN.

**Figure 2:**
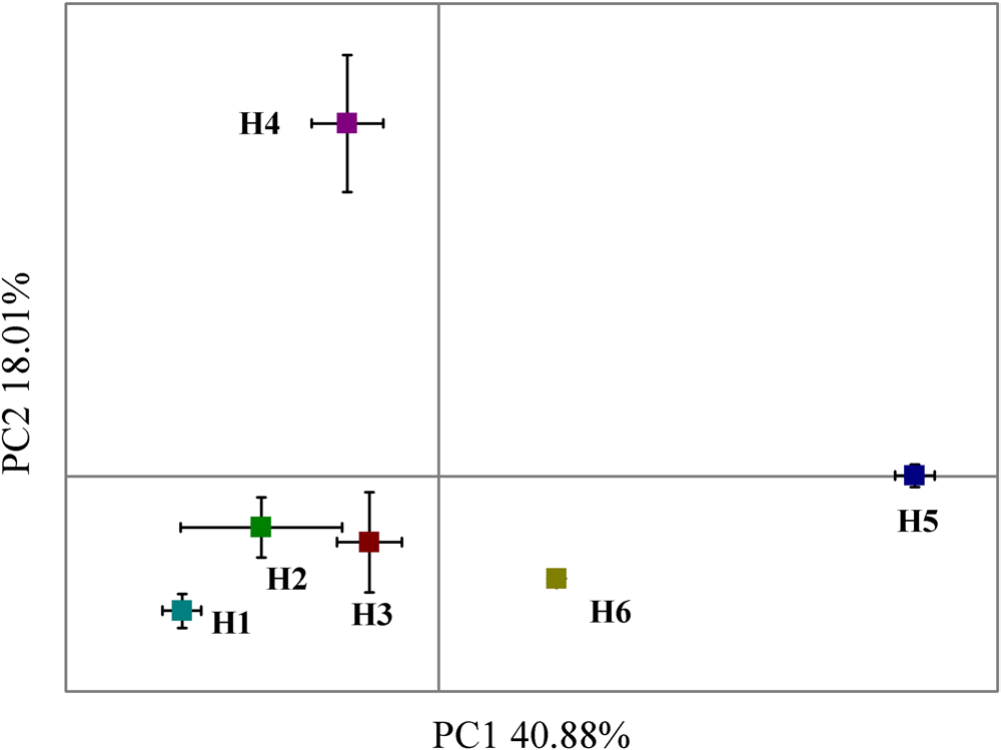
The PCA results of the carbon substrate utilization patterns among the different plots (96 hours).

Generally, three groups of AWCD levels from the Biolog Ecoplate could be partitioned: H5 with the highest utilization efficiency, H1 with the lowest efficiency, and the others with moderate efficiency (Figure S1). A detailed comparison of the different carbon source utilization profiles separated H5 and H6 from the others, and H4 was also separated from H1-H3 (Fig. 2).

### Factors driving the assemblage of the soil microbial community

Soil microbial communities were associated with various environmental factors, including local physicochemical properties and geographical heterogeneity. We tested the correlation between the community profiles and related variables using Pearson correlation, and the significant results are shown in Table 3. Both the soil microbial biomass (MBC/MBN) and microbial carbon/nitrogen were positively correlated with AFDM, TOC, TN and the water level gradients but negatively correlated with SM, TP, pH and hydroperiod. Metabolic profiles showed an inverse relationship compared to the microbial biomass. Most of the enzyme activities were positively correlated with AFDM, TOC, TN, MBC, MBN and the water level gradients but negatively correlated with SM, TP, pH and hydroperiod, except for BG, which seemed to be less affected. Bacterial and fungal TRFs showed a positive correlation with qCO_2_, pH and TP but were negatively correlated with most soil nutrient parameters. The bacterial Shannon index was positively affected by TP and pH but negatively affected by TOC, while the fungal Shannon index was positively affected by NO_3_-N but negatively affected by most soil nutrient parameters. The aboveground vegetation also showed comprehensive relationships with many factors, with the positive factors including MBC, MBN, BX, NAG, PHOS, TOC, AFDM and WH and the negative factors including qCO_2_, SM, pH, NH_4_-N and hydroperiod (Table 3).

**Table 3:** The significantly correlated relationships (Pearson) between the community profiles and variables (p < 0.05).

Characteristics	Profiles	Variables	
		Positively correlated	Negatively correlated
Microbial biomass and respiration	Microbial biomass (MBC/MBN) Microbial carbon/nitrogen	AFDM, TOC, TN, WH	SM, TP, pH, Hydroperiod
	qCO_2_	SM, pH, TP	AFDM, TOC, TN, WH
Soil enzymes	BX/NAG/PHOS	AFDM, TOC, TN, MBC, MBN, WH	SM, pH, TP, hydroperiod
	BG	TOC	TP
Bacterial biodiversity	Taxa	qCO_2_, pH, TP	MBC, MBN, TOC
	Simpson/Evenness	TP	NH_4_-N
	Shannon	TP, pH	TOC
Fungal biodiversity	Taxa	qCO_2_, pH, TP	MBC, MBN, All C/N/P variables
	Simpson	—	All C/N/P parameters, AFDM
	Shannon	NO_3_-N	MBC, All C/N/P parameters, AFDM
	Evenness	—	BG
Vegetation	VS/VD	NBC, MBN, BX, NAG, PHOS, TOC, AFDM, WH	qCO_2_, SM, pH, NH_4_-N, hydroperiod

Abbreviations: AFDM, ash free dry mass; TOC, total organic carbon; TN, total nitrogen; WH: the height above the water level; SM, soil moisture; TP, total phosphate; MBC/MBN, microbial biomass carbon/nitrogen; VS/VD, vegetation species/abundance.

**Figure 3:**
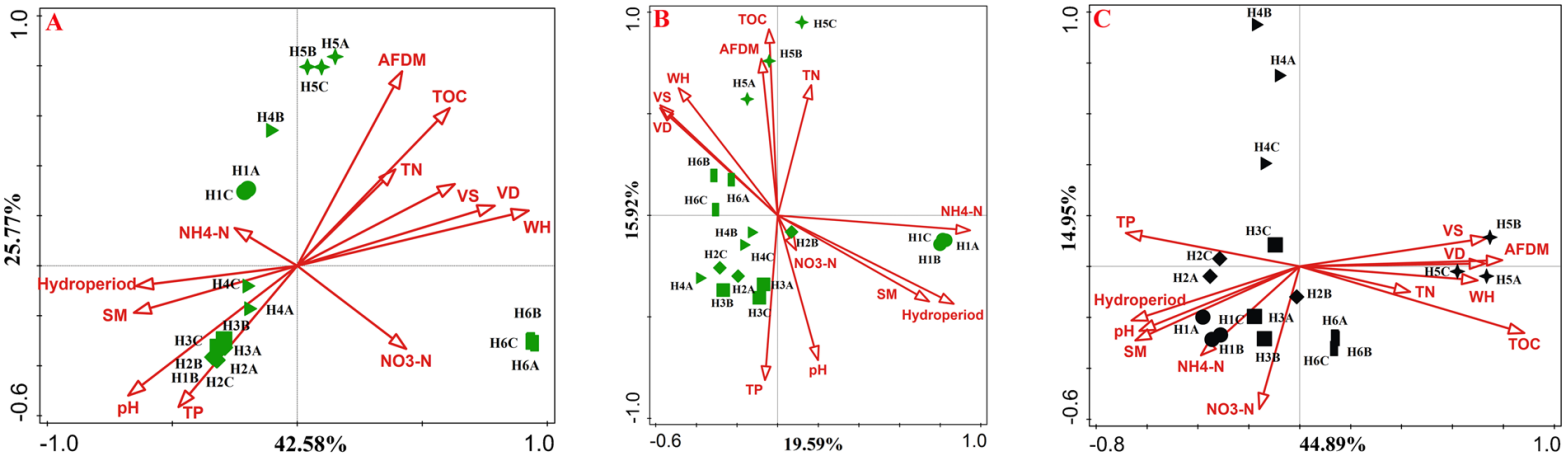
The coordinate analysis between the environmental factors and bacterial communities (**A**), fungal communities (**B**) and carbon utilization profiles (**C**) using the db-RDA method.

The structure and function of the soil microbial communities differed among plots, and many environmental factors contributed to these differences (Fig. 3). For bacteria, the distance-based similarity matrix of the community composition was significantly affected by most of the environmental factors, except for TN, NH_4_-N and NO_3_-N. The water level gradient (WH) was the most significant variable for the H6 plots, while AFDM and TOC were the most significant for the H5 plots and pH and TP for the H2-H4 plots (Fig. 3A). The fungal community patterns were different from the bacterial patterns among the plots, and all the factors except for TN, NH_4_-N and AFDM were significant (*p* < 0.05). The H1 and H5 plots were separated from the other plots. NH_4_-N, SM, and hydroperiod were the most significant contributors to the H1 plot, and AFDM and TOC were the most significant contributors to the H5 plot (Fig. 3B). The carbon utilization profile among the plots was also affected by many environmental factors, but TN, NH_4_-N, NO_3_-N and pH all failed to pass the Monte Carlo test with Bonferroni correction. The H5 and H4 plots were separated from the other plots, and the contribution of AFDM and TOC to the H4 plots was shown (Fig. 3C).

### The discrepancy between the microbial community structure and functional traits in response to the environmental variables

**Figure 4:**
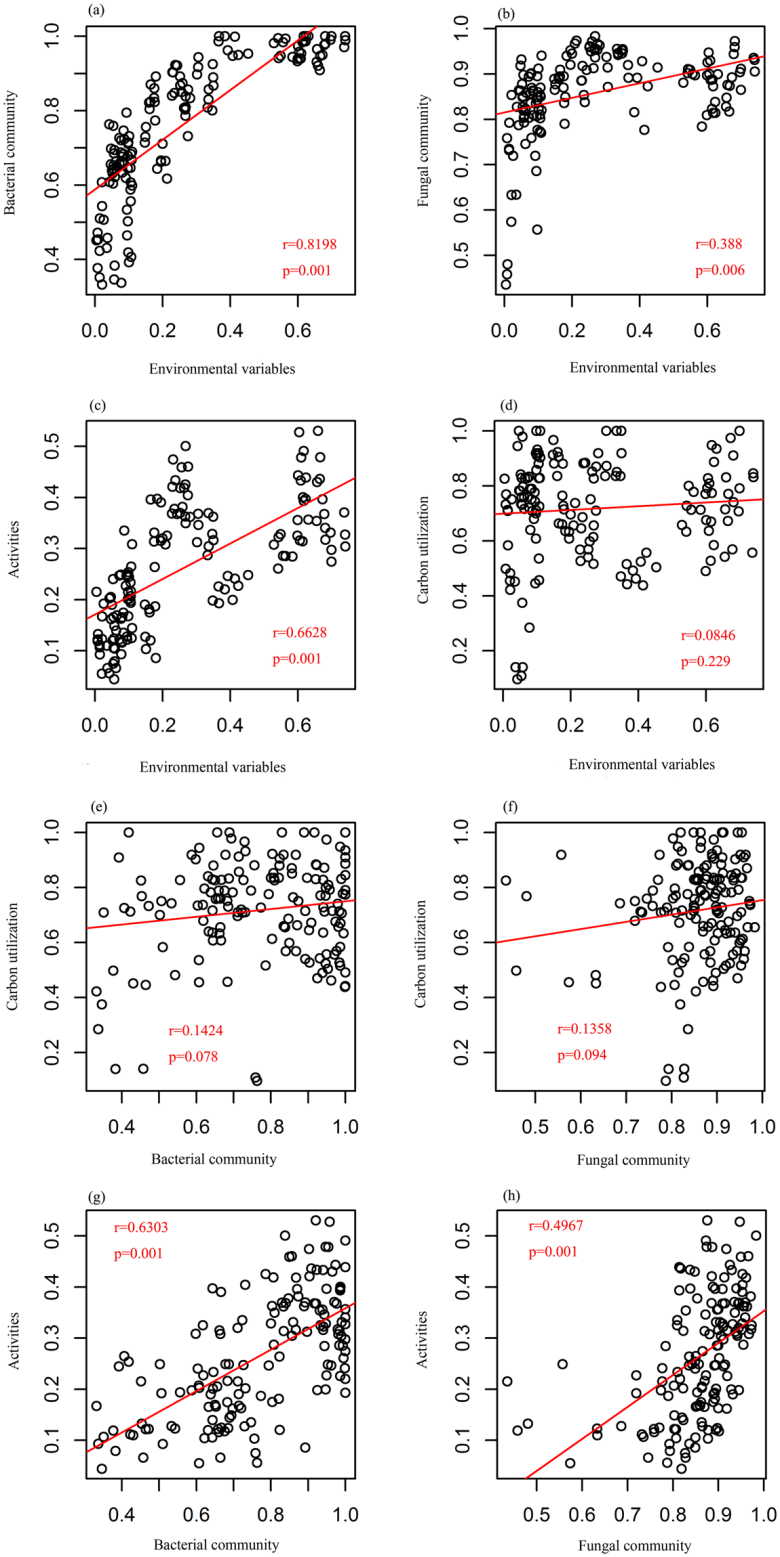
Correlation among environmental heterogeneity, microbial dissimilarity (bacteria and fungi), carbon utilization profiles and soil activities (biomass, enzymes and respiration). Solid lines represent the linear regressions and the significance levels determined by Mantel tests (9999 permutations).

There were differences between the community composition and the functional traits in response to the environmental variables. Environmental variables significantly correlated with both the bacterial and fungal community composition but were not correlated with carbon utilization variables of the soil microbes (Fig. 4a–d). The carbon utilization patterns were so consistent that neither the bacterial nor fungal community structure could be clearly attributed to it (Fig. 4e,f). Soil enzyme activities were correlated with environmental variables and with both the bacterial and fungal community compositions (Fig. 4c,g,h). All the above results represented a distinct discrepancy between the microbial community composition and the functional traits in response to the environmental variables.

### Variance partitioning of the microbial community structures and functional traits

**Figure 5:**
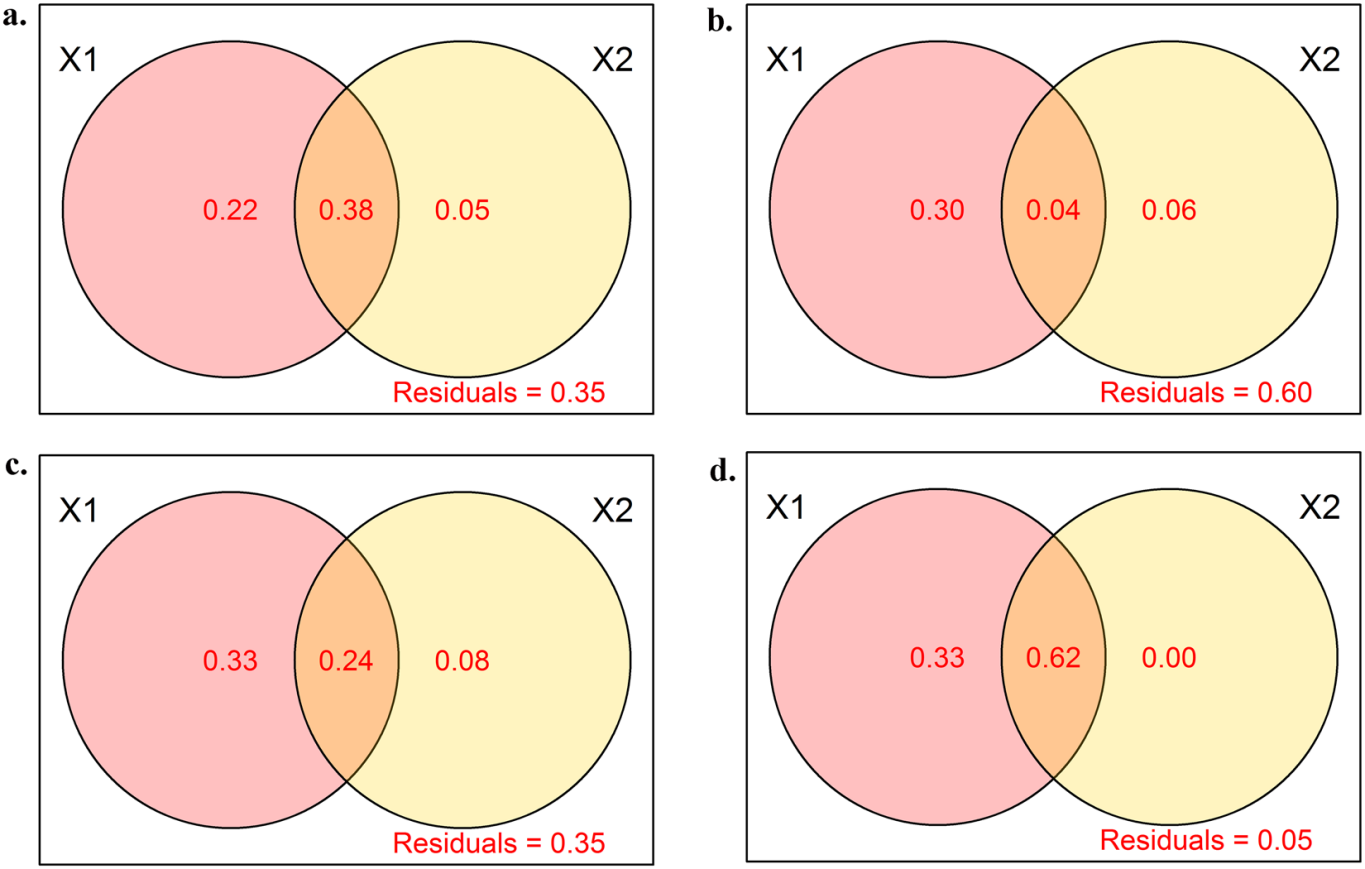
The proportion of variance in the community composition (a for bacterial community, b for fungal community) and functional traits (c for carbon utilization profiles, d for soil activities) explained by the environmental components (X1) and spatial distance (X2). Each diagram represents the variation in a given dissimilarity metric partitioned into the relative effects of each component or combination of components. Unexplained variation (residuals) is marked on each plot.

The variance partitioning method was employed in this study to quantify the relative influence of the stochastic and deterministic processes on the soil bacterial and fungal community compositions as well as on their related functional activity. The combined effect of all the significant environmental variables accounted for 22% and 30% of the total variation in the bacterial and fungal community compositions, respectively, which were larger than the effect of spatial distances, for which 5% and 6% contributions were found, respectively (Fig. 5a,b). For both the bacterial and fungal communities, the combined variables explained less than 40% of the total variation, which implied that other unnoticed factors also contributed to community variation. The environmental effect was also demonstrated to be a dominant player in structuring the functional activity profiles of carbon utilization and soil activities, rather than the spatial distances (Fig. 5c,d). For the carbon utilization profile, the combined environmental factors explained 33% of the total variation, which was higher than the 8% from the spatial effect (Fig. 5c). The profile of the soil activities was mostly governed by the environmental variables (33%) and the related interaction with the spatial distances (62%), while the pure effect of spatial distances was not detected. In addition, the 5% residuals meant that the existing parameters were sufficient to explain the variation in the soil activities (Fig. 5d).

## Discussion

Water fluctuation can strongly affect the lake ecosystem, and wetland soil, as well as the soil microorganisms, always bear the brunt of it. Soil water content varies with water level gradients, and most nutrient elements also exchange with water. Ventilation conditions and redox potentials also shift; thus, the structure and function of the soil microbial community alters. The patterns documented in this study indicate a complex relationship between the microbial community composition and function and note that there is still much to understand regarding the complex relationships between the biotic and abiotic factors.

In this study, soil moisture (SM) gradients were inversely correlated with water level gradients (WH), and the pH values exhibited a unimodal pattern, which was high in the H3 plots. In general, the high SM plots had higher pH values. Both the SM and pH were important factors that affected the soil microbial community composition, and the pH values have been shown to serve as indicators that predict the soil bacterial community^26,32^, while excessive water content in the soil was considered to be a limiting factor for microbial processes^33^. The high SM and pH values in the near water plots illustrated that higher pH values were associated with lake waters, and nitrogen influx into soils of submerged plots was the potential cause for high pH. This pattern was also noted in previous studies that documented water movements transporting nutrients into the sediment in natural freshwater systems^34^. The higher accumulation of AFDM and TOC in higher elevation plots was associated with an increase in the microbial biomass. In contrast to submerged soils, the desiccation process and increased vegetation encouraged the accumulation of organic matter and the greater microbial growth^35^.

All enzymes peaked in the H5 plots of this study, and the carbon utilization activity of H5 also exceeded those of the other plots (Table 2 and Figure S1). This pattern revealed the highest microbial activity in the H5 plots, followed by the H6 plots and H1-H4 plots. This distinctly separated trend was also found in the profiles of microbial community composition and carbon utilization among the H5, H6 and other plots (Fig. 3). Microorganisms can regulate enzyme production according to existing substrates in the environment, and the accumulated plant organic matter satisfied the increased costs of the microbial biomass and enhanced enzyme production in the H5 and H6 plots^36^. Meanwhile, the strong correlation between the fungal community composition and soil enzyme activity may reveal the equal importance of bacteria and fungi in soil activity.

Microbial biomass C/N (MBC and MBN) and microbial C/N ratios (MBC/TOC and MBN/TN) were both higher in the H5 and H6 plots, and relatively higher values were observed in the H1 plots compared to the other three plots (H2-H4). The continual flooding conditions of the H1-H4 plots suppressed the massive growth of the soil microorganism, and the higher biomass in the H1 plots may be associated with the aquatic bacterial communities^37^. Despite the differences in the microbial biomass, basal respiration among the plots was consistent, and the water gradients, as well as microbial biomass, did not greatly influence the soil basal respiration. Although previous studies noted the impact of vegetation on soil basal respiration, this impact was not found in this study^38^. We propose that this stable phase of soil basal respiration was covered up by mixing the susceptible surface layer and stable deep layer of the sampled soil cores, and the cold temperature of the sampling seasons may also contributed to it. Along the water gradients, different succession stages of the microbial communities and vegetation developed, and both were driven by water level fluctuations. Although the microbial carbon metabolic profile seemed less influenced by the environmental gradients, the impact of vegetation was observed in shaping the community structure and function by enhanced nutrient cycling^39–41^. Soil basal respiration, enzyme activities and microbial biomass all showed correlations with the soil properties and microbial community composition, but this finding varied from other reports that documented a slow response of the community composition to environmental changes^42^. The great variation range of the environmental conditions, as well as the regular water regime, were considered to be a major cause of the active response of soil biotic and abiotic variables to water fluctuation in this study^43^.

The soil microbial community structure and function showed different response patterns to water gradients. The community structure seemed more sensitive to water gradients, while the carbon metabolism was more stable (Fig. 4). Compared to fungi, there was a stronger correlation between the bacterial community composition and water level. Soil moisture was documented as a major factor influencing the microbial community structure and enzyme activities in this study (Table 3), and a similar conclusion was obtained from the forest soil samples that also revealed a close relationship between the microbial community composition and soil moisture^27^. Considering the connection between the microbial community composition and microbial activity, the microbial biomass, soil respiration and soil enzymes were all identified as indicators of soil microbial activity^41,44^. Soil microbial activity was correlated with the microbial community structure and showed an active response to environmental changes^45^.

However, for carbon metabolism, no obvious connection was found with either environmental characteristic or microbial community structure (Fig. 4e,f). This independent profile may play important roles in the wetland ecosystem, including maintaining a stable and balanced carbon transformation process and providing a resilient mechanism allowing the ecosystem function to be maintained when facing diverse environmental disturbances. There were two possible explanations for this metabolic independence that we considered. Soil nutrient levels are considered important factors that influence the stability of soil biological functions. This is true especially for TOC, as higher TOC is helpful for maintaining the original function of soil microbial communities in response to drying-rewetting pressures^46^. Another common hypothesis for this independence is functional redundancy. While a small group of functions are regulated by only a small proportion of the microbial community with specialized physiological pathways, the existence of relatively ubiquitous organisms will mask these minor differences in the community structure-function relationships^47–49^.

Simplified variance partition models have revealed the dominance of the deterministic process on the assemblage of the soil microbial community and extensions on soil activity and microbial functions^50^. This result may emphasize the importance of local scale environmental variables and highlight the high variability and complexity of the soil microbial community in wetlands. Environmental properties create abiotic stress on soil microbes and strengthen the interactions among species, which result in more specialized niches. The significant difference among the heterogeneous environmental gradients filtered the microbial composition and hindered the stochastic processes^20,30^.

In conclusion, this study sheds light on the role of the soil microbial process within the function of the wetland ecosystem, documents the response of the microbial community composition and functional activity to water level gradients, and reveals the assemblage processes of bacterial and fungal communities. We also speculate that a decline of the lake water level would lead to stimulated microbial growth and activity and would alter the structure and function of soil microbial communities associated with Poyang Lake wetlands. In addition, lake water shrinkage would induce plant succession towards the lake centre and would strengthen organic matter accumulation and mineralization, but the basal respiration and carbon utilization would remain stable. Increased understanding of the impact of environmental conditions on microbial community composition and functional activity will help us to develop better management strategies in the face of a changing climate.

## Methods

### Study design and sampling procedures

**Figure 6:**
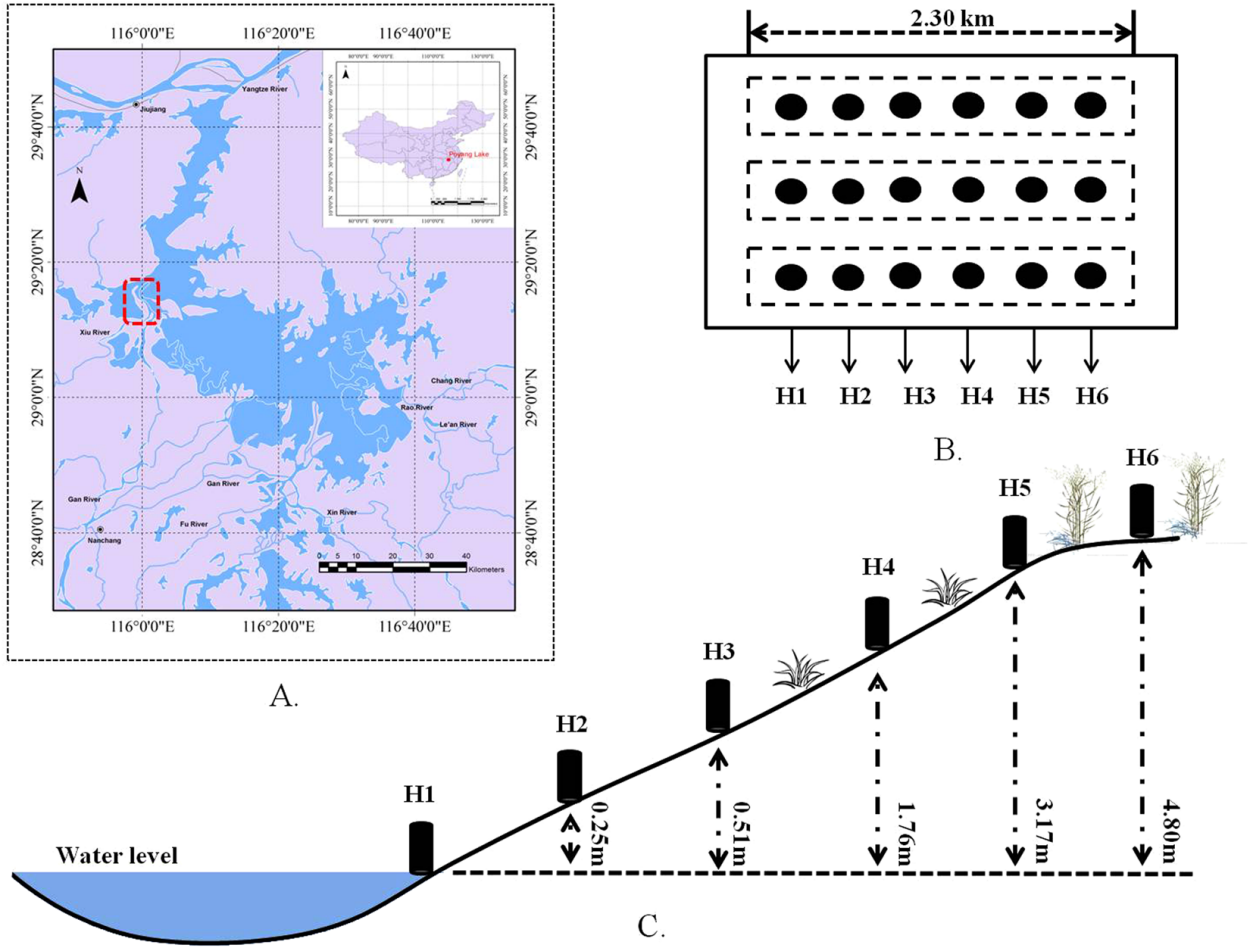
Study field and sampling sites in Lake Poyang of China. The location of the study field in Lake Poyang is shown in (**A**) (generated by ArcGIS 10.2, www.esri.com), and three sampling belts with six plots were placed as shown in (**B**); the vertical distance to the water level is demonstrated in (**C**).

As the biggest freshwater lake of China, Poyang Lake is in the northern part of Jiangxi province, connects to the downstream Yangtze River, and acts as an important reservoir for flood regulation^31^. Due to the high throughput water conditions of Poyang Lake, the water areas have varied dramatically, with an annual water level fluctuation of over 10 metres. Lake wetlands were recorded as internationally important wetland habitats by the United Nations in 1992^7^. In recent years, the water regime of Poyang Lake has changed, resulting in reduced water levels (hydroperiod) and extended dry seasons. Banghu Lake, a dish-shaped sublake of Poyang Lake, was selected as the field site for this study. Banghu Lake was sensitive to water level changes, and the riparian vegetation varied according to site elevations^51^ (Fig. 6A).

We selected three transects along a shore-side wetland area. Each transect (500 m apart) had six plots (H1-H6, 10 m*10 m) for sampling (shown in Fig. 6B and C). The H1 plot was the lowest water point that was continually submerged in lake water, and the other plots were distributed along the wetland slope with increased elevation. The H1 and H2 plots were bare without any vegetation, the H3 and H4 plots had sparse, small plants, and the H5 and H6 plots were covered by lush forbs and graminoids.

Surface soil samples (0–15 cm) were collected in March 2013 (this time of year has the lowest yearly water level and the broadest shoreline as well as the ideal water gradients) with an aseptic soil sampler. A total of 10 independent soil cores (2.5 cm in diameter) were randomly collected within each plot and pooled as one sample. Samples collected from the 18 plots were transferred to the laboratory in an ice-cold box. In the lab, each of the soil samples were subdivided into two parts, one part for physicochemical analysis and Biolog Ecoplate assay and the other part for molecular analysis (stored at −80 °C). Vegetation species and abundance were investigated by measuring the number of species (vegetation species) and strains (vegetation abundance) of several 1 m2 areas for each plot.

### Physicochemical analysis

We measured the soil moisture (SM), pH, ash free dry mass (AFDM), total organic carbon (TOC), total nitrogen (TN), total phosphate (TP), ammonia nitrogen (NH_4_-N) and nitrate nitrogen (NO_3_-N) of all the collected samples (Table 1). The detailed measuring methods for the above parameters were described in a previous study^31^. NH_4_-N and NO_3_-N were extracted from 10 g of wet soil using a 2 M KCl extraction procedure and then measured by a discrete auto analyser (Smartchem 200, Westco, France)^10^.

### DNA extraction and T-RFLP analysis

Total DNA was extracted from 0.5 g soil samples using a PowerSoil DNA isolation kit (MoBio, USA) following the manufacturer’s instructions. T-RFLP analysis was performed after the amplification of the 16 S or 18 S rRNA gene fragments and the enzymatic digestion of the PCR products. The primer pairs of Eub338F/1392 R and ITS1F/ITS4 were used for amplification, and the former primer 338 F and ITS1F were both modified with the 6-FAM label^52,53^. The resulting enzymatic fragments were purified and sent to Sangon Biotech Co. Ltd. (Shanghai) for testing. The resulting T-RFLP profiles were normalized by peak height, and only the fragments with a relative abundance over 0.5% were used for community analysis^54^.

### Soil microbial activity and functional traits

#### Soil enzyme analysis

A microplate fluorometric assay method was followed to determine the soil enzyme activity of β-glucosidase (BG), β-D-xylosidase (BX), acid phosphatase (AP) and N-acetyl-β-D-glucosaminidase (NAG)^55^. Generally, 1 g of soil was added to 125 ml of 50 mM acetate buffer (pH 5.0) and homogenized; then, the sample suspensions were mixed with fluorogenic substrates in 96-well microplates and incubated in the dark at 25 °C for 0.5–2 h. Ten microlitres of 0.5 M NaOH was added to each well to stop the reaction, and the microplates were examined in a Varioskan Flash (Thermo Scientific, USA) with 365 nm excitation and 450 nm emission filters. After correcting for the negative controls and quenching, all the tested enzyme activities were expressed in units of μmol h^−1^ g^−1^ dry soil^56^.

### Soil microbial biomass and basal respiration (BR) analysis

All soil samples were stored at 4 °C and measured within three days^57^. Soil samples were sufficiently homogenized after removal of any visible plant roots or stones, and the microbial biomasses of the carbon and nitrogen (MBC/MBN) were determined following the standard chloroform-fumigation approach^58^.

Soil basal respiration (BR) was determined by quantifying the carbon dioxide (CO_2_) released from microbial respiration during 10 days of incubation. Generally, 25 g soil samples were sealed in a confined space with NaOH (0.5 M) to capture the released CO_2_, and the amount of CO_2_ was determined by titration with 0.5 M HCl, with barium chloride (0.25 M BaCl_2_) and phenolphthalein added before titration. The respiration rate was calculated as the average CO_2_ content respired hourly per kilogram soil^59^.

### Carbon utilization analysis

Heterotrophic microbial activity from the wetland soils was measured as the carbon utilization patterns by Biolog Ecoplate (Hayward, USA)^60^. Approximately 10 g of soil samples (dry weight equivalent) as dispersed in 90 ml of aseptic normal saline and then diluted 100 times, and 150 μl of diluent was added into each well of the plate. The original optical density (OD) was measured immediately with an automated plate reader (BIOLOG Inc., USA) at 590 nm. After a 25 °C incubation every 12 hours in a constant temperature humidity chamber, the OD was documented, and the procedure lasted up to 10 days to ensure the carbon utilization saturation phase was reached in all the samples^61^.

### Statistical analyses

Statistical analysis of the environmental variables and soil activity parameters among the sampling plots was determined using one-way ANOVA by the SPSS 21.0 software package. The soil microbial community dataset, which was characterized by T-RFLP profiles, was analysed using PAleontological Statistics (PAST) Version 3.11 for community diversity^17^. Hierarchical clustering analysis was used for the soil microbial community, and the Bray-Curtis dissimilarity matrix of abundance based on RFs was calculated in the R project 3.3.1 (“ape” and “vegan”) with the single method^14^. All the ordination analysis was done with Canoco 5.0, and the models of PCA and db-RDA were used in this study^43^. Pearson correlation analysis between the biological observations and environmental variables was assessed in SPSS 21.0. Generalized Linear Models (GLM) and Mantel tests (Pearson’s correlation) in the R project were used for the relationship analysis among the microbial community (bacteria and fungi), environmental variables, geographic distance and vegetation^14^. To further estimate the relative importance of the deterministic and stochastic processes on the community assemblage of the microbial structure and function, variation partitioning was performed in R with a distance-based approach as previously described^62^.

## Electronic supplementary material

Supplementary Material
