# Supplementary material for: Bacterial and Fungal Community Composition and Functional Activity Associated with Lake Wetland Water Level Gradients
Source: Sci Rep. 2018 Jan 15;8:760. doi: 10.1038/s41598-018-19153-z (PMC5768796; doi:10.1038/s41598-018-19153-z)
Supplement: Supplementary file 1 — Supplementary Material [file 41598_2018_19153_MOESM1_ESM.pdf]

Supplementary Material for

Bacterial and Fungal Community Composition and Functional Activity Associated with Lake Wetland Water Level Gradients

Yantian Ma[1,2#], Jinqian Li[1#], Juan Wu[1], Zhaoyu Kong[1], Larry M. Feinstein[3], Xia Ding[1], Gang Ge[1], Lan Wu[1,2*]

[1]*Key Laboratory of Poyang Lake Environment and Resource, Ministy of Education, and School of Life Sciences, Nanchang University, Nanchang 330022, China*

[2]*Key Laboratory of Aquatic Animal Resources and Utilization of Jiangxi, Nanchang University, Nanchang 330022, China*

[3] *University of Maine at Presque Isle, Presque Isle, Maine, USA*

* Corresponding authors:
L. Wu, ncusk724@hotmail.com

This file includes

Table S1

Figure S1

Table S1.The community diversity of soil bacteria and fungi from this study using the T-RFLP method.

Plots	Bacteria				Fungi			
	Taxa	Simpson	Shannon	Evenness	Taxa	Simpson	Shannon	Evenness
**H6**	29.33±2.08**a**	0.90±0.01**bc**	2.81±0.10**bc**	0.57±0.01**c**	35.33±10.50**ab**	0.92±0.03**b**	2.99±0.40**b**	0.58±0.06**bc**
**H5**	26.67±5.51**a**	0.86±0.01**a**	2.47±0.17**a**	0.45±0.02**a**	26.33±6.35**a**	0.86±0.03**a**	2.52±0.35**a**	0.48±0.04**ab**
**H4**	31.67±2.52**ab**	0.89±0.03**ab**	2.71±0.18**ab**	0.48±0.05**ab**	37.67±1.15**b**	0.91±0.02**b**	3.01±0.10**b**	0.54±0.07**abc**
**H3**	37.33±2.52**b**	0.92±0.002**c**	3.07±0.04**c**	0.58±0.02**c**	36.33±7.02**ab**	0.90±0.01**b**	2.95±0.20**b**	0.53±0.002**abc**
**H2**	33.00±7.00**ab**	0.89±0.02**abc**	2.79±0.24**bc**	0.50±0.02**b**	38.33±0.58**b**	0.90±0.004**ab**	2.90±0.02**ab**	0.47±0.003**a**
**H1**	29.33±4.93**a**	0.86±0.03**a**	2.57±0.23**ab**	0.45±0.03**a**	35.00±1.00**ab**	0.91±0.01**b**	2.96±0.03**b**	0.55±0.01**bc**

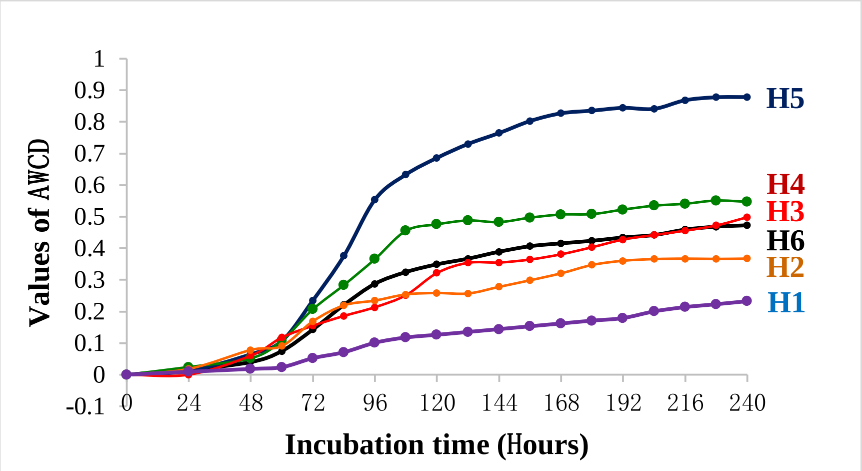

Figure S1. The values of AWCD (Average Well Color Development) from different samples with Biolog Eco-plate method.
